# Rapid and simultaneous detection of human hepatitis B virus and hepatitis C virus antibodies based on a protein chip assay using nano-gold immunological amplification and silver staining method

**DOI:** 10.1186/1471-2334-5-53

**Published:** 2005-07-06

**Authors:** Lianlian Duan, Yefu Wang, Shawn Shun-cheng Li, Zhixiang Wan, Jianxin Zhai

**Affiliations:** 1 Department of Biotechnology, College of Life Sciences, Wuhan University, 430072, Wuhan, Hubei, People's Republic of China; 2 Department of Biochemistry, University of Western Ontario, London, Ontario N6A 5C1, Canada

## Abstract

**Background:**

Viral hepatitis due to hepatitis B virus and hepatitis C virus are major public health problems all over the world. Traditional detection methods including polymerase chain reaction (PCR)-based assays and enzyme-linked immunosorbent assays (ELISA) are expensive and time-consuming. In our assay, a protein chip assay using Nano-gold Immunological Amplification and Silver Staining (NIASS) method was applied to detect HBV and HCV antibodies rapidly and simultaneously.

**Methods:**

Chemically modified glass slides were used as solid supports (named chip), on which several antigens, including HBsAg, HBeAg, HBcAg and HCVAg (a mixture of NS3, NS5 and core antigens) were immobilized respectively. Colloidal nano-gold labelled staphylococcal protein A (SPA) was used as an indicator and immunogold silver staining enhancement technique was applied to amplify the detection signals, producing black image on array spots, which were visible with naked eyes. To determine the detection limit of the protein chip assay, a set of model arrays in which human IgG was spotted were structured and the model arrays were incubated with different concentrations of anti-IgG. A total of 305 serum samples previously characterized with commercial ELISA were divided into 4 groups and tested in this assay.

**Results:**

We prepared mono-dispersed, spherical nano-gold particles with an average diameter of 15 ± 2 nm. Colloidal nano-gold-SPA particles observed by TEM were well-distributed, maintaining uniform and stable. The optimum silver enhancement time ranged from 8 to 12 minutes. In our assay, the protein chips could detect serum antibodies against HBsAg, HBeAg, HBcAg and HCVAg with the absence of the cross reaction. In the model arrays, the anti-IgG as low as 3 ng/ml could be detected. The data for comparing the protein chip assay with ELISA indicated that no distinct difference (P > 0.05) existed between the results determined by our assay and ELISA respectively.

**Conclusion:**

Results showed that our assay can be applied with serology for the detection of HBV and HCV antibodies rapidly and simultaneously in clinical detection.

## Background

The hepatitis B virus (HBV) and hepatitis C virus (HCV) often cause persistent infection, leading to chronic liver diseases, cirrhosis and hepatocellular carcinoma [1,2]. Given the burden of these diseases and the current potential for cure, there is a compelling need for diagnosis of active HBV and HCV infection. A variety of HBV and HCV markers have been used to detect HBV and HCV infection. Gene amplification tests, such as PCR-based [3-7] assays are used to diagnose and monitor the efficacy of treatment. However, these methods require cumbersome procedures and expensive equipment, thus requiring considerable skills and high costs. Immunoassays are generally easy and inexpensive. So far, some immunological methods such as enzyme-linked immunosorbent assays (ELISA) and rapid diagnostic paper have been used in clinical practice. While the value and significance of these methods are beyond argument, they suffer from several disadvantages, mainly their inability to produce results simultaneously. Ruo-Pan Huang [8] has detected multiple cytokines and antibodies simultaneously on nitrocellulose membrane, utilizing horseradish peroxidase (HRP)-conjugated antibodies as detecting reagents and visualizing the signals with an enhanced chemiluminescence (ECL) system. However, this method is time-consuming and requires expensive set-up, limiting its large-scale application. Mezzasoma *et al*. [9] have detected serum antibodies against the TORCH antigens on amino-silane-activated glass slides with fluorescently labelled secondary antibodies. Unfortunately, this method is also limited in clinical applications due to the cost of the assay.

In the past few years, protein chip and microarray technology has shown its great potential in the functional analysis of the proteome, clinical diagnostics and drug discovery. It allows fast, easy and parallel detection of thousands of addressable elements in a single assay. For instance, the potential of this technology to diagnose human diseases, such as leukemia, breast cancer and, potentially, heart failure, has stimulated much interest. In our previous studies, we established a platform on which gene chips with a high sensitive visual detection based on two-probe sandwich hybridization/nanoparticle amplification have been employed, and HBV and HCV gene fragments were detected on a glass slide by visual inspection [10,11]. In this paper, we developed a protein chip technology based on NIASS method. A protein chip was devised to detect antibodies of HBV and HCV easily and simultaneously. In this assay, the enhancing solution was the physical developer that contained both silver ions and a reducing agent, buffered to an acidic pH. During silver enhancement, the colloidal nano-gold served as a nucleation site for the deposition of metallic silver and the particles grew in size, giving an intensely dark signal which could be visualized with the naked eyes. Colloidal nano-gold labelled SPA was used as a detecting reagent which could bind specifically to the Fc portion of immunoglobulin from many mammals. The clinical performance of this assay was validated with a collection of serum samples previously characterized with commercial ELISA for their reactivity against the selected antigens. The data displayed that no distinct difference (P > 0.05) existed between the results determined by our assay and ELISA respectively. In a preliminary test, our assay detected up to 3 ng/ml anti-IgG, which was close to that in the fluorescent detection method.

## Methods

### Preparation of nano-gold particles

Colloidal nano-gold solutions were prepared by the citrate reduction of HAuCl_4 _according to the literature [12], filtered through a 0.45 μm nylon filter, and stored at 4°C. Prior to use, all glassware was immersed in cleaning solution (200 g potassium dichromate and 500 ml of concentrated sulfuric acid dissolved in distilled water to the volume of 2000 ml) for 24 hours, rinsed 3 times with ddH_2_O and then dried in oven. A transmission electron microscope (TEM, HITACHI H-8100, Japan) was used to determine the size of the colloidal nano-gold particles and UV-Vis spectrum was used to analyze the uniformity of the nano-gold particles.

### Determination of the optimum ratio of colloidal nano-gold solution to SPA concentration

Prior to the preparation of colloidal nano-gold labelled SPA, the optimum ratio of colloidal nano-gold solution to SPA concentration should be determined. Colloidal nano-gold solution was centrifuged (500 g) for 15 minutes, and the precipitate was eliminated. Serial volume SPA solution (0.05 mol/L) was added to 1000 μl of the colloidal nano-gold solution which had been adjusted to pH 5.5 with 0.2 M potassium carbonate, followed by incubation for 15 minutes at room temperature. After 100 μl of 10% (w/v) NaCl solution was added to the mixture, the absorbance at 520 nm was measured, and the SPA solution volume showing maximum absorbance was regarded as the optimum. The volume of SPA solution in practical operation generally increases 10%–20%.

### Preparation of nano-gold-SPA probes

Thirteen microlitres of a SPA solution was incubated with 1000 μl of the colloidal nano-gold solution (pH 5.5) for 15 minutes at room temperature, followed by addition of 5% (w/v) bovine serum albumin (BSA) solution to a final concentration of 1%. The mixture was allowed to stand for over 15 minutes, and was centrifuged (50000 g, 4°C) for 45 minutes twice. After each cycle, the supernatant was eliminated and the red precipitate was washed with 20 mmol/L Tris buffer (pH6.5, filtrated through a 0.22 μm membrane) including 1% (w/v) BSA and 0.02% (w/v) NaN_3_. Following the final cycle, the precipitate was suspended in the same buffer and stored at 4°C.

### Preparation of the protein chip

Super-flat optic glass slides (BAIO, China) were prepared according to the literature [13]. Briefly, the slides were soaked in 10% (w/v) NaOH and then 0.1 M HCl for 2 hours, respectively. After a thorough rinsing with ddH2O and boiling for 1 hour, the glass slides were set in 1% (v/v) 3-glycidoxypropyltrimethoxysilane (GOPTS) solution (GOPTS dissolved in 95% ethanol) at 37°C for 6 hours, followed by drying and incubating at 135°C for 1 hour, and finally stored at 4°C.

Nought point one microlitres of the antigen solution (100 μg/mL) was manually printed onto the chemically derivated slide. Arrays included four antigens (HBsAg, HBeAg, HBcAg and HCVAg which was a mixture of NS3, NS5 and core antigens) printed in four replicates. SPA and HIVEnv36 were used as positive and negative control respectively. All the antigens were diluted with phosphate-buffer saline (PBS, 137 mmol/L NaCl, 2.7 mmol/L KCl, 10 mmol/L Na_2_HPO_4_, 2 mmol/L KH_2_PO_4_, adjusted to pH7.4 with HCl) with 40% glycerol to the final concentration. In the assay of evaluating the detection limit of the protein chip technology, SPA, human IgG and HIVEnv36 (100 μg/mL) were spotted onto the GOPTS-activated slides in four replicates to prepare a set of model arrays.

The slides were placed at 37°C with the humidity maintaining at 90% for 1 hour, followed by rinsing three times with PBS to remove the unbound proteins.

### Blocking of the protein chip

The chip was immersed in 1% (w/v) BSA (dissolved in PBS) solutions for 30 minutes with gentle shaking, rinsed three times with PBST solution (Tween-20 dissolved in PBS to a final concentration of 0.05%) and finally rinsed twice with PBS.

### Detection of antibodies in patients' sera

Forty microlitres of the colloidal nano-gold-SPA solution was mixed with 10 μl of serum sample. The mixture was diluted with PBS buffer, bringing the total volume to 1000 μl, and incubated for 30 minutes at 37°C. The mixed solution was placed on the surface of the chip for 30 minutes at 37°C with the humidity maintaining at 90%. The chip was rinsed three times with PBS, dried and covered with silver enhancer solution, which was composed of 700 μl of citric acid buffer (2.55 g citric acid and 2.35 g sodium citrate dissolved in 1000 ml of ddH_2_O, pH3.5), 300 μl of hydroquinone solution (1.7 g hydroquinone dissolved in 30 ml of ddH_2_O) and 20 μl of silver nitrate solution (2.5 mg silver nitrate dissolved in 100 μl of ddH_2_O). After treatment with the silver enhancer solution, the chip was rinsed with water, and then air-dried.

### Data collection and analysis

The slides were analyzed with a scanner (FOUNDER, China) and the density of each spot was determined by the software taken by the scanner.

To compare the protein chip assay with ELISA, a total of 305 serum samples showing different activities in the ELISA (low, medium and high) collected from Xiehe Hospital (Wuhan, China) were detected. The use of the serum samples was approved by the patients. The sera were divided into 4 groups (Table 1) and each sample was assayed individually. The sera were stored at -4°C and previously characterized with commercial ELISA (KHB, China), which were performed according to the manufacturer's instructions. The results were collected and analyzed.

**Table 1:** The division of serum samples for detection

	HBsAb	HBeAb	HBcAb	HCVAb
Group-1(n = 90)	+	-	-	-
Group-2(n = 85)	-	+	+	-
Group-3(n = 80)	-	-	-	+
Group-4(n = 50)	-	-	-	-

## Results

### Size and configuration of colloidal nano-gold and nano-gold-SPA

A TEM was used to determine the size distribution of the colloidal nano-gold particles. The resulting particles remained stable and formed uniform spheres. At least 200 particles were measured with TEM, of which the average diameter was 15 ± 2 nm.

**Figure 1:**
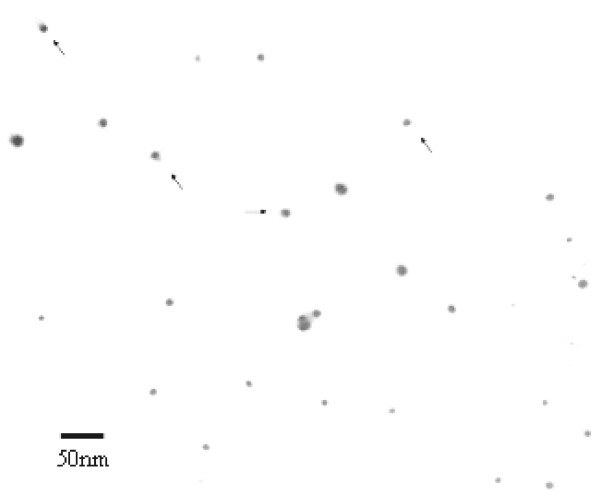
**Transmission electron micrograph of nano-gold-SPA probes**. The arrowheads pointed to nano-gold-SPA probes. The structure of halo disturbance surrounding the nano-gold was SPA proteins. In keeping the objective of reacting with antibodies, SPA was chosen, for that, SPA could be site-specifically attached to Fc region of antibody, preserving its antigen-combining activity. And nano-gold clustered for silver enhancement.

The so prepared nano-gold-SPA particles were stained by phosphate tungstate and observed by TEM, shown in Figure 1. Colloidal nano-gold particles surrounded by SPA were still well-distributed, maintaining uniform and stable.

### Comparison of UV-Vis spectra between colloidal nano-gold and nano-gold-SPA

**Figure 2:**
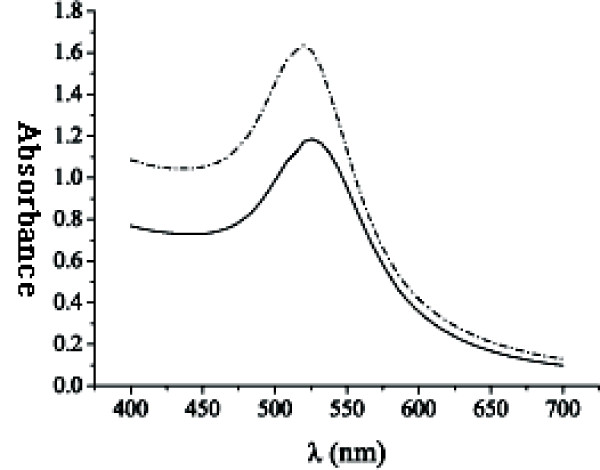
**Comparison of UV-Vis spectra between colloidal nano-gold and nano-gold-SPA probes**. The dash dot curve denoted UV-Vis spectrum of colloidal nano-gold within the range of 400-700 nm. The solid curve denoted UV-Vis spectrum of colloidal gold-SPA probes within the range of 400-700 nm. A(gold)/520 was 1.636920, whereas A(gold-SPA)/520 was 1.185927, and the loss of colloidal nano-gold was about 27.55%, which was due to centrifugation during preparation of nano-gold-SPA probes.

**Figure 3:**
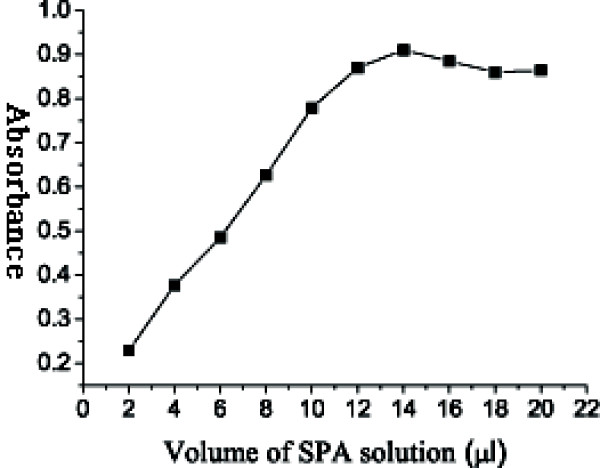
**Determination of ratio between colloidal nano-gold and SPA**. The value of abscissa denoted the volume of SPA solution added into each tube of colloidal nano-gold solution, while the value of ordinate denoted O.D value of each tube at the wavelength of 520 nm. The O.D value increased with the adding of SPA solution, and reached the maximum when colloidal nano-gold was saturated by SPA.

A UV-Vis spectrophotometer (UNICO™, China) was employed to scan the absorbance of colloidal nano-gold in the range of 500-550 nm with a scanning precision of 1 nm. Only one peak was displayed in the scanning curve (figure not shown), which indicated that so prepared colloidal nano-gold was uniform. This method was also used to scan the absorbance curve of colloidal nano-gold and colloidal nano-gold-SPA in the range of 400-700 nm with a scanning precision of 6 nm. The results were shown in Figure 2, in which the peak wavelength was 520 nm, whereas that of nano-gold-SPA was 526 nm. Still only one peak was displayed in the scanning curve. The shift of λ_max_was not due to the surface modification of colloidal nano-gold, but to the change in gold particle distribution during centrifugation of colloidal nano-gold-SPA. It meant that λ_max _of colloidal nano-gold didn't shift along with the mutual reaction between nano-gold and SPA. The optimum ratio of colloidal nano-gold solution to SPA concentration was determined in the wavelength of 520 nm. According to the Figure 3, the optimum volume of SPA solution was about 12-14 μl (0.05 mol/L).

### The effects of various silver enhancement time on signal intensity (relative density)

Immunogold silver staining enhancement technique was applied to amplify the detection signals, which greatly enhanced the sensitivity of the assay. Therefore, the results could be visualized by the naked eyes because the small nano-gold particles could easily be grown to a useful size.

**Figure 4:**
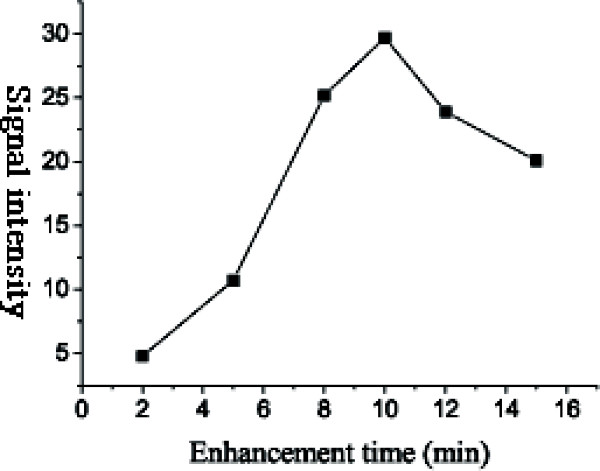
**The effects of various silver enhancement time on signal intensity**. The concentration of antigens spotted on the slides was 100 μg/ml. The silver enhancement time varied from 5 to 15 minutes. The result indicated the optimum time for silver development ranged from 8 to 12 minutes. Both too short (less than 5 minutes) and too long (more than 15 minutes) development might bring on darkened background.

The data in Figure 4 indicated that the detected results were related to the silver enhancement time. The optimum time ranged from 8 to 12 minutes. When the time exceeded 15 minutes, the background darkened in a decrease in signal intensity which was determined by both spot intensity and background intensity.

### Detection limit of the protein chip assay

**Figure 5:**
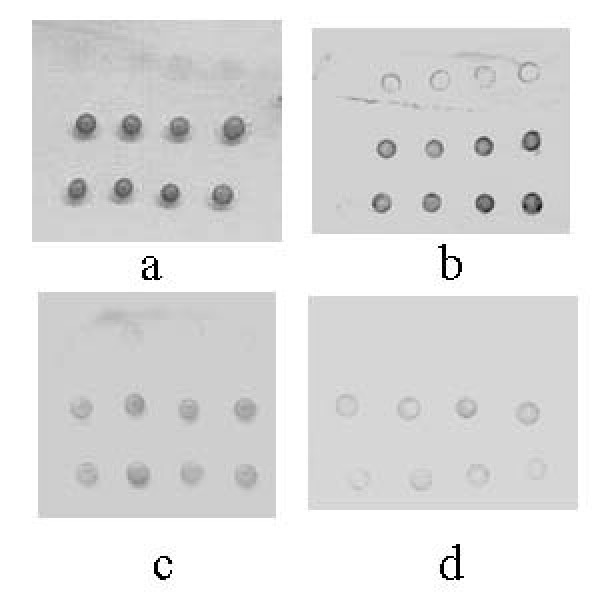
**Detection limit of the protein chip assay**. a: The concentration of anti-IgG was 3 μg/ml. b: The concentration of anti-IgG was 300 ng/ml. c: The concentration of anti-IgG was 30 ng/ml. d: The concentration of anti-IgG was 3 ng/ml. The first, second and third row denoted the negative control, detection and positive control spots respectively. The concentration of human IgG spotted on the slides was 100 μg/ml. The silver enhancement time was 10 minutes.

The detection limit of the protein chip assay was demonstrated by incubation of model arrays with different concentrations of anti-IgG. The experiment was for guidance only. As shown in Figure 5, the anti-IgG as low as 3 ng/ml could be detected, such a sensitivity was close to that in the fluorescent detection method (1 ng/ml) [14].

### Comparison of protein chip assay and ELISA

The results for comparison of the protein chip assay with ELISA were shown in Table 2, 3 and 4. Paired chi-square test indicated that no distinct difference (P > 0.05) existed between the results determined by our assay and ELISA respectively. While with ELISA formats, each analyte was detected in a separate assay, thereby increasing time and costs.

**Table 2:** Comparison of the protein chip assay with ELISA test protocols (Group-1 and Group-4 serum samples)

Protein chip assay	ELISA		Total samples
		
	HBsAb(+), HBeAb(-), HBcAb(-), HCVAb(-)	HBsAb(-), HBeAb(-), HBcAb(-), HCVAb(-)	
HBsAb(+), HBeAb(-), HBcAb(-), HCVAb(-)	86	0	86
HBsAb(-), HBeAb(-), HBcAb(-), HCVAb(-)	4	50	54

P > 0.05

**Table 3:** Comparison of the protein chip assay with ELISA test protocols (Group-2 and Group-4 serum samples)

Protein chip assay	ELISA		Total samples
		
	HBsAb(-), HBeAb(+), HBcAb(+), HCVAb(-)	HBsAb(-), HBeAb(-), HBcAb(-), HCVAb(-)	
HBsAb(-), HBeAb(+), HBcAb(+), HCVAb(-)	80	0	80
HBsAb(-), HBeAb(-), HBcAb(-), HCVAb(-)	5	50	55

P > 0.05

**Table 4:** Comparison of the protein chip assay with ELISA test protocols (Group-3 and Group-4 serum samples)

Protein chip assay	ELISA		Total samples
		
	HBsAb(-), HBeAb(-), HBcAb(-), HCVAb(+)	HBsAb(-), HBeAb(-), HBcAb(-), HCVAb(-)	
HBsAb(-), HBeAb(-), HBcAb(-), HCVAb(+)	75	0	75
HBsAb(-), HBeAb(-), HBcAb(-), HCVAb(-)	5	50	55

P > 0.05

**Figure 6:**
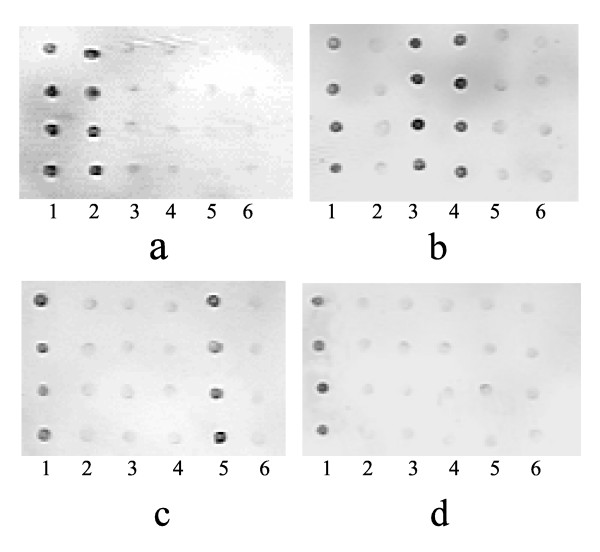
**The demonstration of protein chips for detection of serum samples**. a: The chip for detection of serum sample from Group-1 b: The chip for detection of serum sample from Group-2 c: The chip for detection of serum sample from Group-3 d: The chip for detection of serum sample from Group-4 Line 1: positive control spots Line 2: HBsAg spots Line 3: HBeAg spots Line 4: HBcAg spots Line 5: HCVAg spots Line 6: negative control spots The concentration of each protein spotted on the slides was 100 μg/ml. The silver enhancement time was 10 minutes.

Furthermore, no reactivity was detected against HBeAg, HBcAg, HCVAg and negative control in Group-1, against HBsAg, HCVAg and negative control in Group-2, against HBsAg, HBeAg, HBcAg and negative control in Group-3, and against HBsAg, HBeAg, HBcAg HCVAg and negative control in Group-4, thus indicating the absence of cross reaction in the assays (Figure 6).

## Discussion

The preparation of colloidal nano-gold-SPA was a key process of the entire experiment. Many different routes to produce colloidal nano-gold particles have been reported [15-19]. The vast majority of processes involved the reduction of gold compounds in both aqueous and nonaqueous media. In our assay, we applied a versatile precipitation method capable to generate mono-dispersed, spherical nano-gold particles using aqueous solution of gold chloride and sodium citrate as the reducing agent. During the manipulation of preparing the nano-gold-SPA, pH of the gold solution was carefully controlled. Excessively acidic or basic conditions could easily cause the nano-gold-SPA to deposite. Colloidal nano-gold particles carried positive charges at acidic condition, allowing attachment of SPA through steric interaction. Therefore, adjusting pH to or above the isoelectric point of SPA could increase negative charges on SPA surface. In our assay, Tris buffer was undertaken to store the nano-gold-SPA. And the stability of nano-gold-SPA solution could be destroyed at room temperature or frozen condition. Four centigrade was becoming to store the nano-gold-SPA solution.

Actually silver enhancement reaction processes rapidly. Silver ions in solution nucleate around nano-gold particles and precipitate as silver metal. Particle grows in size with time of development. It was of importance to control the reaction time because both too short and too long development might bring on darkened background. Besides, the distribution of nano-gold on the chip and the uniformity of nano-gold played a part in the enhancement result.

Protein A is a cell wall component of *Staphylococcus aureus *that binds specifically to the Fc portion of immunoglobulin from many mammals [20,21]. And the binding does not interfere with antigen-antibody reaction. Also SPA can bind to colloidal nano-gold through static interaction, forming the labelling probes. In our assay, the well-prepared colloidal nano-gold-SPA could be preserved for over 1 year, keeping stable and active. Compared with horseradish peroxidase-conjugated antibodies and fluorescently labelled secondary antibodies [8,9], the colloidal nano-gold-SPA was more economical and convenient, thus reducing the cost of the assay and simplifying the manipulation of the experiment.

The common supports used for immobilization are either glass microscope slides or membranes; however, glass is preferred because it is more amenable to automation and exhibits a lower background signal [22]. To attach proteins to a solid substrate, the surface of the substrate has to be modified to achieve the maximum binding capacity. Coating the glass surface with poly-L-lysine (PLL) is a convenient method which is also applied in DNA microarrays [23]. The attached proteins are passively adsorbed to the surface in random orientation through non-specific interactions and can be washed off under stringent washing conditions [24]. There are several methods available for the surface activation of glass slides, many of which have been described [25-33]. Most methods introduced a chemical group onto the surface of the glass and then react with active groups of protein molecules. In our assay, the glass slides were activated by GOPTS. The preparation of the slides was convenient while the immobilization efficiency was high. The use of the chemically derivated slides coupled with printing solutions whose concentrations were optimized in our assay allowed us to generate visible results suitable for a study aimed at assessing the clinical performance of the protein chip assay.

We compared the detection results tested by the protein chip assay and ELISA, respectively. In Group-1, 4 samples were detected as HBsAb negative. This might be due to the very low concentration of HBsAb in serum samples which could not be detected by the protein chip assay. The same applied to Group-2 and Group-3. Paired chi-square test indicated that no distinct difference (P > 0.05) existed between the results determined by the two methods. While the protein chip assay could detect in parallel antibodies on one chip without non-specific reactions, thus it is more convenient and costs less time than ELISA. Moreover, it required small quantity of both samples and reagents, which made it economical and useful for those conditions when large quantities of samples were not easy to get. Furthermore, it is cheaper and safer than the fluorescent and isotope ones. The well-prepared protein chip could be preserved for over 1 year without losing of activity.

## Conclusion

The protein chip assay described is sensitive and specific, easy to perform, and could provide results in less than 40 minutes. In addition, several kinds of antibodies could be detected simultaneously without cross reaction. The results of this study suggest that there is potential for the application of our method in clinical diagnosis of various infectious diseases.

## Competing interests

The author(s) declare that they have no competing interests.

## Authors' contributions

Lianlian Duan and Yefu Wang were responsible for conception and design of the study. Lianlian Duan collected all data and wrote first draft of the manuscript. Zhixiang Wan and Jianxin Zhai were responsible for statistical analysis. Yefu Wang supervised the study and together with Shawn Shun-cheng Li participated in design and critical review of the manuscript. All authors have read and approved the manuscript.

## Pre-publication history

The pre-publication history for this paper can be accessed here:


